# Postpubertal-Type Teratoma of the Retroperitoneal Space as a Late Recurrence of a Testicular Germ Cell Tumor: A Case Report With Clinical Insight

**DOI:** 10.7759/cureus.71667

**Published:** 2024-10-16

**Authors:** Afag Aslanova, Masanori Ishida, Reisuke Takahashi, Yutaro Suzuki, Elly Arizono, Yukari Wakabayashi, Yoshio Ohno, Toshitaka Nagao, Kazuhiro Saito

**Affiliations:** 1 Radiology, Tokyo Medical University Hospital, Tokyo, JPN; 2 Pathology, Tokyo Medical University Hospital, Tokyo, JPN; 3 Urology, Tokyo Medical University Hospital, Tokyo, JPN

**Keywords:** late recurrence, mixed germ cell tumor, non-seminoma, postpubertal-type teratoma, retroperitoneal tumor, seminoma, testicular germ cell tumor

## Abstract

Postpubertal-type teratomas are rare malignant tumors derived from germ cell neoplasia in situ (GCNIS). This case report presents a rare instance of a retroperitoneal postpubertal-type teratoma as a late recurrence of a testicular germ cell tumor (GCT) that was initially diagnosed as a seminoma. A 48-year-old male who had undergone left inguinal orchidectomy for a testicular mass was diagnosed with a seminoma (stage I) six years prior and presented with an asymptomatic 6-cm retroperitoneal tumor near the left renal hilum. Initial blood tests at presentation for the retroperitoneal tumor were normal, except for a mild elevation of lactate dehydrogenase. Computed tomography (CT), magnetic resonance imaging, and fluorine-18-fluorodeoxyglucose positron emission tomography (18F-FDG PET)/CT imaging revealed a well-defined tumor with calcification and high glucose metabolic activity. A CT-guided biopsy for the retroperitoneal tumor suggested a high-grade malignant tumor of neuroendocrine origin. The patient underwent neoadjuvant chemotherapy, which resulted in tumor shrinkage and decreased metabolic activity. Subsequent surgical resection and histopathological examination revealed a postpubertal-type teratoma with a concomitant neuroendocrine tumor. A reexamination of the previous testicular tumor specimen revealed a small amount of embryonal carcinoma within the seminoma, indicating that the initial diagnosis should have been a mixed germ cell tumor rather than a pure seminoma. This explains the unusual recurrence pattern observed and highlights the importance of a thorough histological examination for testicular GCTs, as microscopic non-seminomatous components can significantly affect prognosis and recurrence patterns. When encountering suspected recurrences of GCNIS-derived GCTs, clinicians should consider the possibility of an initially undetected mixed GCT, particularly in cases with atypical presentation or recurrence patterns.

## Introduction

According to the 2016 and 2022 WHO classification systems, testicular germ cell tumors (GCTs) are classified into two categories: a group derived from germ cell neoplasia in situ (GCNIS) and a non-GCNIS group. Tumors derived from GCNIS may be classified into seminoma and non-seminomatous germ cell tumors (non-SGCT). The non-SGCT group includes embryonal carcinoma, postpubertal-type yolk sac tumor, postpubertal-type teratoma, trophoblastic tumors, and mixed GCTs [1].

Of the testicular GCTs, teratomas may be divided into two types: a prepubertal type, which is non-GCNIS-related and child-onset, and a postpubertal type, which is of GCNIS origin and adult-onset. All cases of the postpubertal type are malignant tumors. Pure teratomas are frequently identified in metastases after chemotherapy for non-SGCTs, which often contain teratomatous elements in the testis [2]. Postpubertal-type teratoma can be expressed as a metastasis of non-SGCT derived from the GCNIS; however, even advanced seminomas rarely contain teratomas in the residual tumor after treatment [3], and it is not reasonable to consider stage I seminomas recurring with teratomas.

Here, we present a case of a patient who underwent an orchidectomy for a testicular tumor and was diagnosed with a seminoma at the time and found to have a retroperitoneal tumor six years following surgery. We present the pathology of the case based on the interpretation of pathological findings of previous testicular tumors and a newly discovered retroperitoneal tumor. Through this case with an atypical course, the pathological and clinical pitfalls that can occur in cases diagnosed as stage I seminoma are presented, and clinical insights are given on how to deal with these.

## Case presentation

A 48-year-old male patient with no medical comorbidities presented to our hospital. Six years earlier, he underwent left inguinal orchidectomy for a testicular mass, which proved to be seminoma based on histology (pT1; confined to the testis and no lymphovascular invasion) and stage I based on the postoperative diagnosis. He recovered well, had no major complaints, and underwent follow-up computed tomography (CT) scans for one year without any evidence of recurrence. The patient was lost to follow-up but revisited a tertiary care hospital six years later at the recommendation of his primary care physician. The patient was asymptomatic, and blood tests, including alpha-fetoprotein, human chorionic gonadotropin, free beta human chorionic gonadotropin, carcinoembryonic antigen, and soluble interleukin-2 receptor, were normal except for a mildly elevated lactate dehydrogenase of 333 U/L (normal range: 124-222 U/L) (Table 1).

**Table 1:** Blood test results at the revisit There was a mild elevation in lactate dehydrogenase levels, while the other blood test results were normal.

Parameters	Value	Normal range
White blood cell	7600/μL	3300–8600 /μL
Neutrophil	69.0%	42.0–74.0%
Lymphocytes	19.0%	19.0–47.0%
Monocytes	7.1%	2.0–8.0%
Eosinophil	4.2%	<6.0%
Basophil	0.7%	<2.0%
Red blood cell	494 x 10,000 /μL	435 x 10,000 to 555 x 10,000 /μL
Hemoglobin	14.8 g/dL	13.7-16.8 g/dL
Platelets	293 x 1,000 /μL	140 x 1,000 to 340 x 1,000 /μL
Total protein	7.2 g/dL	6.6–8.1 g/dL
Albumin	4.3 g/dL	4.1–5.1 g/dL
Total bilirubin	0.67 mg/dL	0.4–1.5 mg/dL
Aspartate aminotransferase	32 U/L	13–30 U/L
Alanine aminotransferase	17 U/L	10–42 U/L
Lactate dehydrogenase	333 U/L	124–222 U/L
Alkaline phosphatase	41 U/L	38–113 U/L
Gamma-glutamyl transpeptidase	37 U/L	13–64 U/L
Blood urea nitrogen	20.0 mg/dL	8.0–20.0 mg/dL
Creatinine	0.79 mg/dL	0.65–1.07 mg/dL
Sodium	139 mEq/L	138–145 mEq/L
Potassium	4.4 mEq/L	3.6–4.8 mEq/L
Chlorine	102 mEq/L	101–108 mEq/L
Calcium	9.3 mEq/L	8.8–10.1 mEq/L
C-reactive protein	0.12 mg/dL	<0.30 mg/dL
Alpha-fetoprotein	5.1 ng/mL	<10.0 ng/mL
Human chorionic gonadotropin	<2.0 mIU/mL	<9.9 mlU/mL
Free beta human chorionic gonadotropin	<0.1 ng/mL	<0.1 ng/mL
Carcinoembryonic antigen	<2.0 ng/mL	<5.0 ng/mL
Soluble interleukin-2 receptor	357.0 U/mL	122–496 U/mL

**Figure 1:**
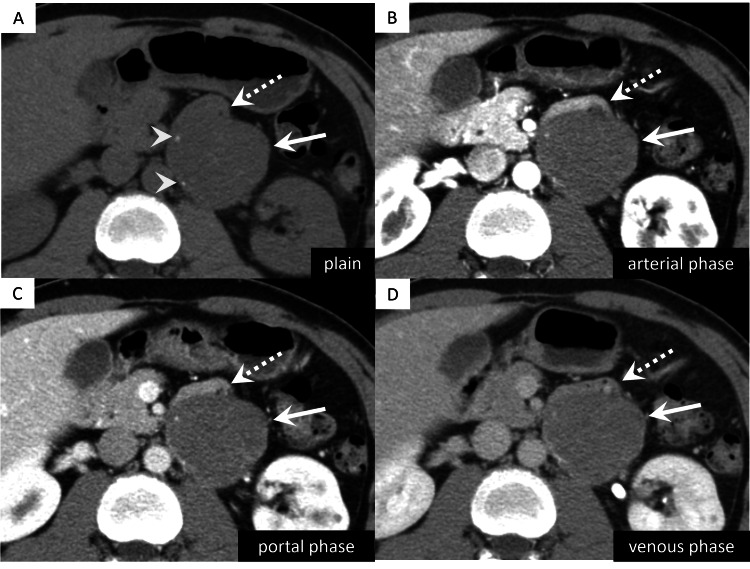
Abdominal computed tomography imaging findings (A-D) Computed tomography (CT) images showing a 6-cm, well-defined tumor near the left renal hilum (arrows). The fourth part of the duodenum located ventral to tumor (dotted arrows). (A) Plain CT scan showing small calcifications at the right border of the mass (arrowheads). (B-D) Contrast-enhanced CT images showing a persistent mild enhancement in both the arterial and portal venous phases with extension into the venous phase.

Abdominal computed tomography (CT) scan with intravenous contrast revealed a 6-cm, well-defined retroperitoneal tumor near the left renal hilum (Figure 1). A standard CT scan revealed a small calcification at the right border of the mass. Dynamic contrast-enhanced CT showed a persistent mild enhancement in the arterial and portal venous dominant phases with extension into the venous phase.

**Figure 2:**
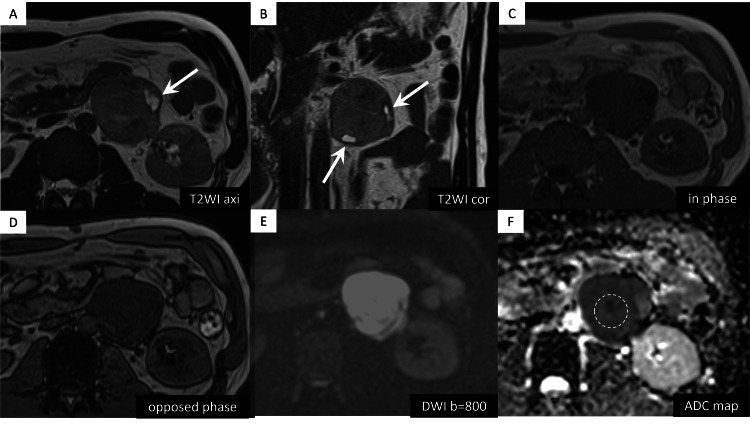
Abdominal magnetic resonance imaging findings (A-F) A mass showing mild hyperintensity on T2-weighted images (T2WI), low signal intensity on T1-weighted images (dual gradient-echo in-phase and opposed-phase). (A,B) T2WI revealed a high-signal area within part of the mass, suggesting the presence of degenerative or necrotic changes (arrows). There was no evidence of a hemorrhagic or fat component. (E,F) Marked diffusion restriction was observed on diffusion-weighted imaging (DWI) and the measurement of the region of interest placed for apparent diffusion coefficient (ADC) map was 0.86 × 10^−3^ mm^2^/s (dotted circle).

Abdominal non-contrast magnetic resonance imaging (MRI) revealed low signal intensity on the T1-weighted image, mild hyperintensity on the T2-weighted image, and diffusion restriction (apparent diffusion coefficient value = 0.86 × 10−3 mm2/s) (Figure 2). There was no signal suggesting a hemorrhagic or fat component.

**Figure 3:**
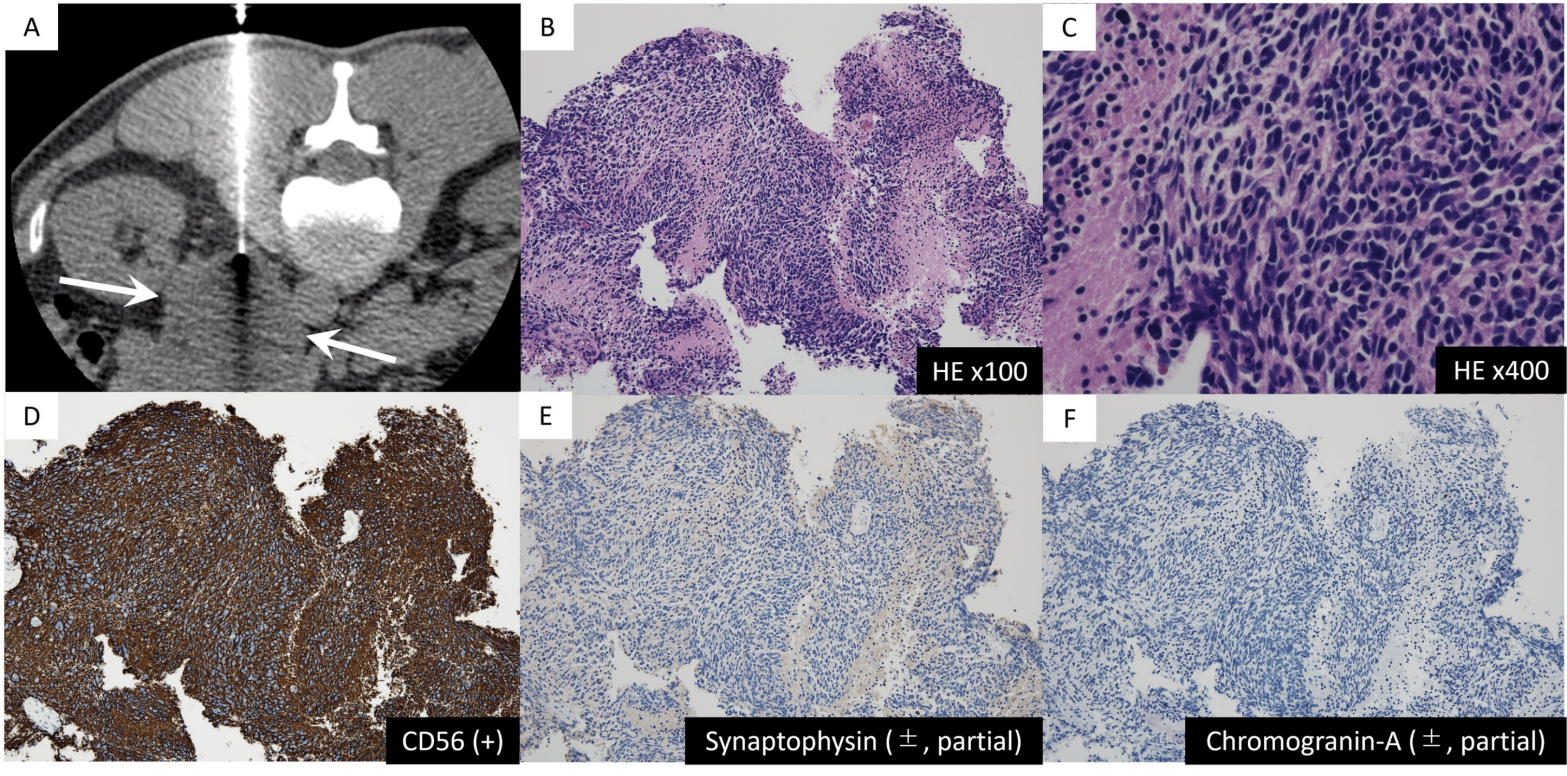
Specimen obtained by computed tomography-guided needle biopsy for the left retroperitoneal mass (A) Computed tomography-guided biopsy for left retroperitoneal mass (arrows) in the prone position. (B) Hematoxylin and eosin (HE) staining showing undifferentiated tumor cells arranged in nests and sheets. (C) High-power magnification of the HE stained specimen showing advanced nuclear atypia and slight nuclear mitosis, which confirmed highly active tumor tissue. (D-F) Immunostaining was positive for CD56 (stained brown), with some positive staining of synaptophysin and chromogranin A (stained light brown). These findings are consistent with the diagnosis of a malignant tumor with neuroendocrine transformation.

Fluorine-18-fluorodeoxyglucose positron emission tomography (18F-FDG PET)/CT was also performed, and the maximum standardized uptake value (SUVmax) was 15.6. Based on the radiological findings, a late relapse of testicular seminoma to the lymph nodes or primary retroperitoneal tumor was suspected; however, because the previous testicular tumor was a pT1 seminoma with a rare late recurrence, we decided to perform a tumor biopsy to obtain a definitive diagnosis. A CT-guided needle biopsy was performed. Hematoxylin and eosin staining of the tissue revealed undifferentiated tumor cells arranged in nests and sheets (Figure 3). High-power magnification revealed advanced nuclear atypia and slight nuclear mitosis, which confirmed highly active tumor tissue. Immunostaining revealed positivity for CD56, with partial positive staining for synaptophysin and chromogranin A. The pathological diagnosis of the tumor was “high-grade malignant tumor of neuroendocrine origin,” which was not compatible with the previous testicular seminoma.

**Figure 4:**
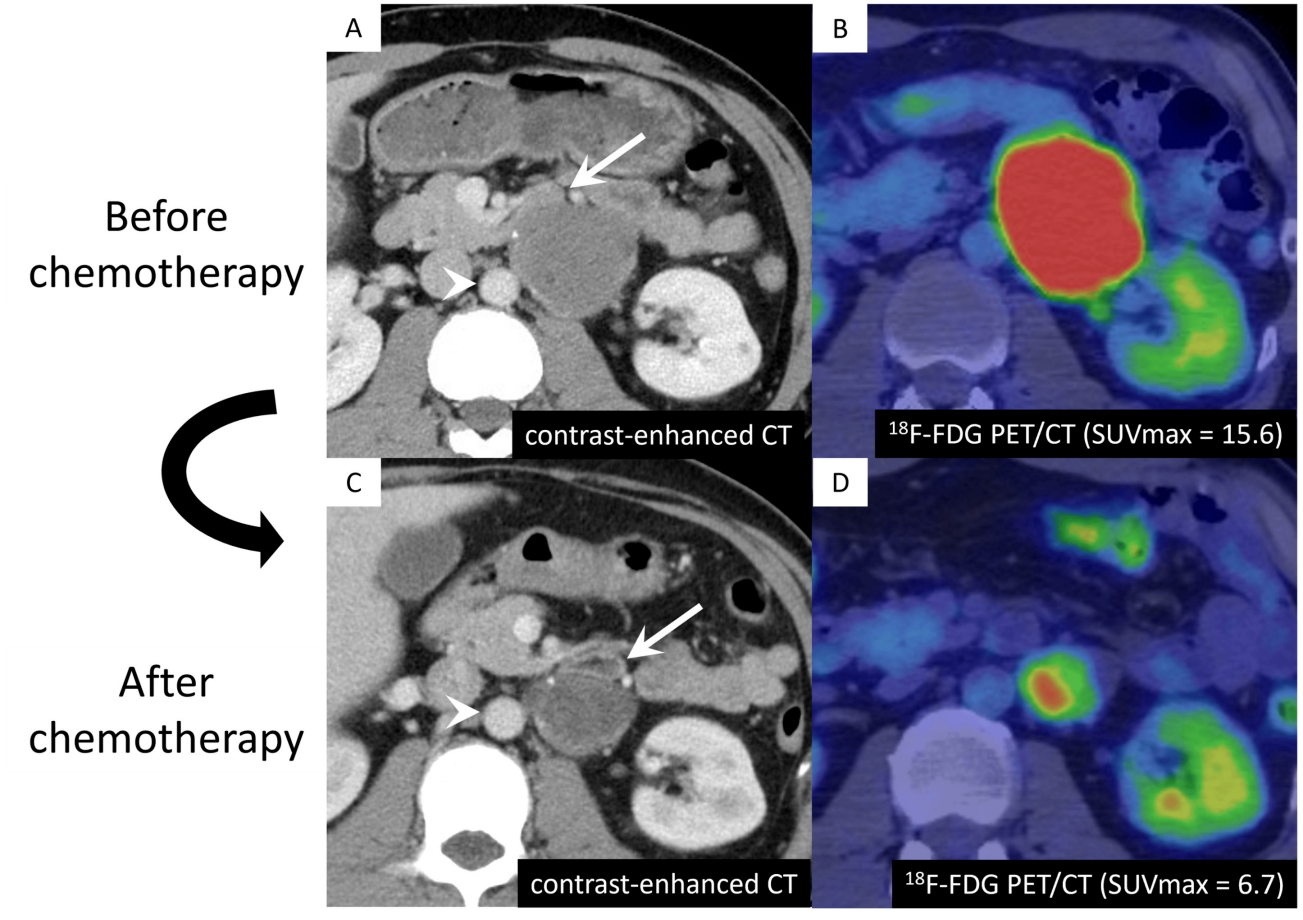
Preresected tumor before and after chemotherapy The patient underwent treatment for a retroperitoneal mass suggestive of a neuroendocrine tumor. (A,B) Because the mass was in contact with the pancreas, duodenum (arrows), and aorta (arrowheads) on contrast-enhanced computed tomography (CT), the patient was treated with adjuvant chemotherapy to reduce the tumor size before resection. (C,D) On the fluorine-18-fluorodeoxyglucose positron emission tomography (^18^F-FDG PET)/CT, the maximum standardized uptake value (SUVmax) values decreased from 15.6 to 6.7 following chemotherapy.

Before resection, the patient underwent neoadjuvant chemotherapy (cisplatin and etoposide) with a regimen similar to that of small cell carcinoma to reduce the tumor size. Following chemotherapy, the size of the tumor decreased from 6 cm to 4.2 cm based on a CT scan, and the SUVmax values from a 18F-FDG PET/CT decreased from 15.6 to 6.7 (Figure 4).

**Figure 5:**
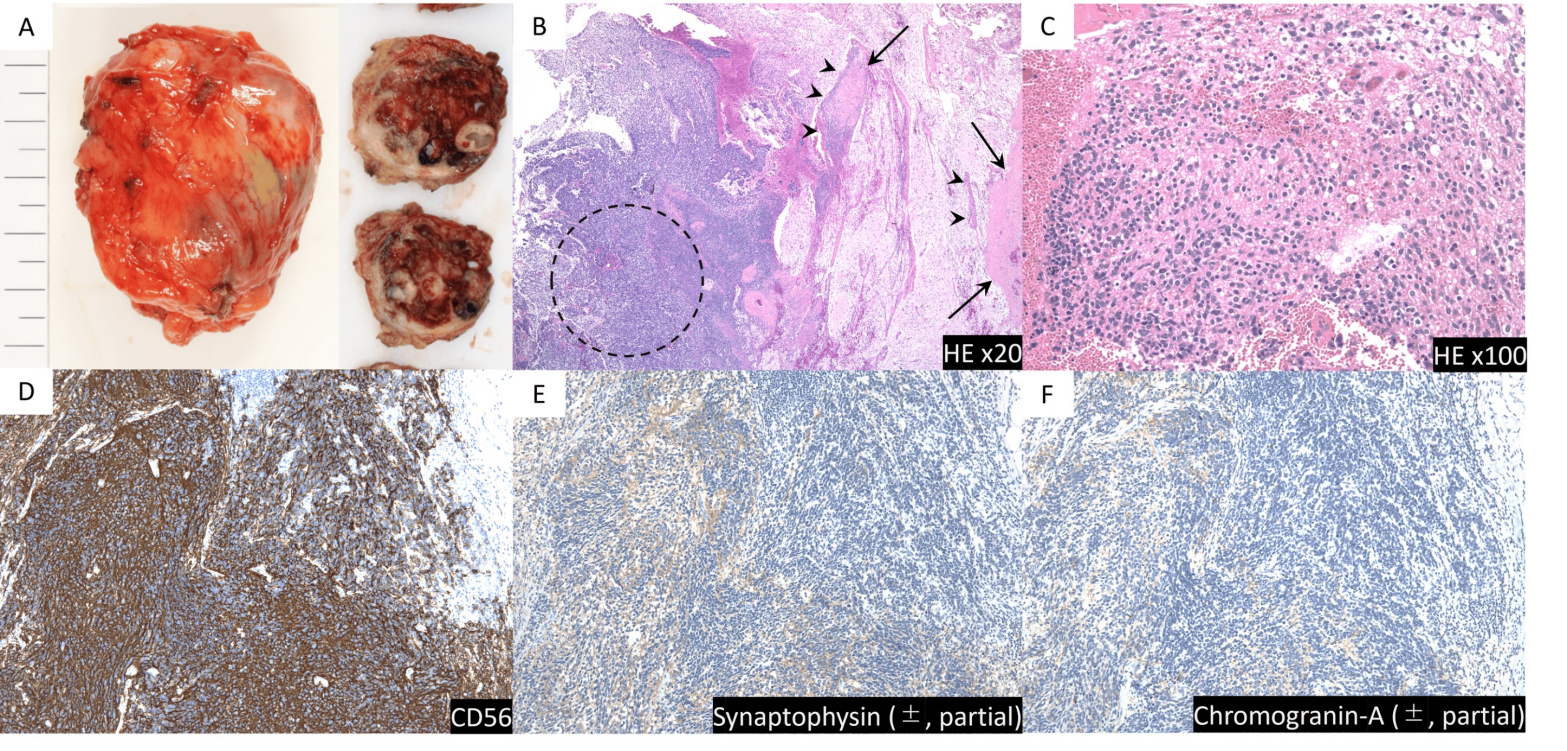
Histopathology of the excised tumor (A) The excised tumor was covered in a capsule with no invasion into the surrounding area. A cross-section of the gross specimen revealed heterogeneity, which is a finding suggestive of multiple tissues. (B) Hematoxylin and eosin (HE) staining showing nerve-derived tumor tissue (dotted circle) containing cartilaginous tissue (arrows) and stratified ciliated columnar epithelium (arrowheads) indicating a teratoma. (C) High-power magnification of the HE stained specimen showing neurogenic tissue combined with poorly differentiated neuroendocrine tissue. (D-F) Immunostaining was positive for CD56 (stained brown) with some positive staining for synaptophysin and chromogranin A (stained light brown). The pathological diagnosis of the excised tumor was a postpubertal-type teratoma.

The patient subsequently underwent complete resection of the retroperitoneal mass. A cross-section of the gross specimen revealed heterogeneity, suggesting the presence of multiple tissues (Figure 5).

**Figure 6:**
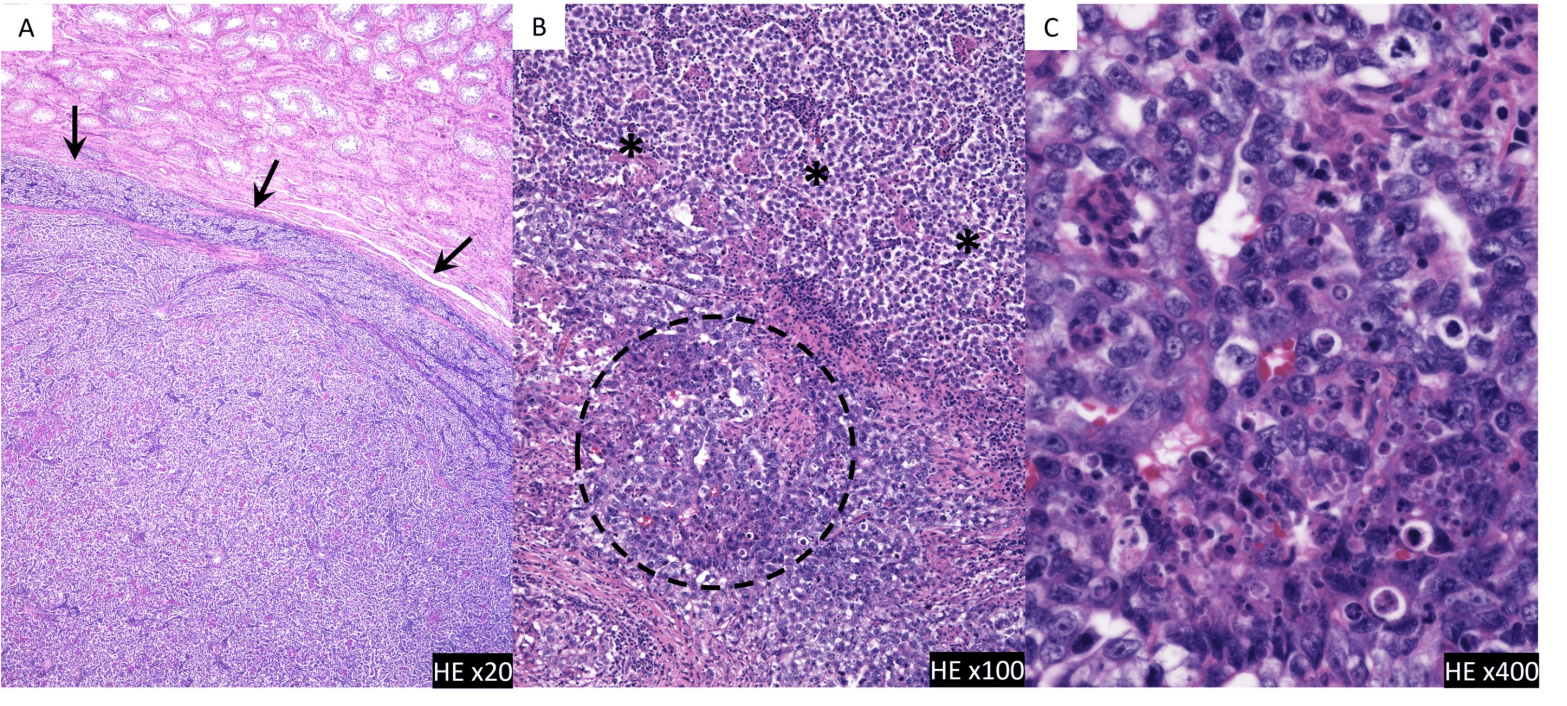
Pathological specimen of the previously resected testicular tumor (A) The specimen shows tumor cells with a high N/C ratio and round nuclei proliferating solidly, which suggests a seminoma (arrows). (B) After excision of the retroperitoneal tumor, the previous testicular tumor specimen was reexamined by a pathologist, who discovered that there was a small amount of embryonic carcinoma (dotted circle) within the seminoma (asterisks). (C) High-power magnification of the hematoxylin and eosin (HE) stained specimen showing the embryonal carcinoma, characterized by pleomorphic cells with glassy nuclei and prominent nuclei. Cellular borders are indistinct.

Histopathological examination revealed primarily nerve-derived tumor tissue containing cartilaginous and stratified ciliated columnar epithelium, which indicated a teratoma. The pathological diagnosis of the excised tumor was “postpubertal-type teratoma” with a concomitant neuroendocrine tumor. There was a tumor component with neuroendocrine differentiation, but no obvious epithelial carcinoma or sarcoma morphology could be identified, and a teratoma with somatic-type malignancy was not diagnosed. Based on the final diagnosis of the retroperitoneal tumor, the previously resected testicular tumor specimen was reexamined by a pathologist. A small amount of embryonic carcinoma within the seminoma, indicating a non-SGCT, was discovered (Figure 6).

It was then clarified that this diagnosis is more appropriate than seminoma. To date, the patient has been followed through regular CT scans for two years without a recurrence of the tumor.

## Discussion

We present a case of a retroperitoneal tumor, which was diagnosed as a postpubertal-type teratoma. The pathological reexamination of a previously resected testicular tumor revealed embryonal carcinoma, which indicated that this postpubertal-type teratoma was a recurrence of a mixed GCT of GCNIS origin.

The development of teratomas in GCT recurrence, as observed in this case, underscores the complex nature of these tumors. This phenomenon is likely related to the pluripotent nature of embryonal carcinoma cells, which can differentiate into various tissue types. Recent studies have highlighted the role of epigenetic alterations, particularly DNA methylation changes, in the progression and recurrence of germ cell tumors [4,5]. Furthermore, genetic aberrations such as isochromosome 12p have been associated with an increased risk of recurrence and may contribute to the development of teratomatous elements. Late recurrence, as seen in our patient, may be attributed to dormant tumor cells that evade initial treatment while retaining the capacity for malignant transformation [6]. The neuroendocrine differentiation observed in this case exemplifies the potential for GCTs to undergo somatic-type malignant transformation, a process that likely involves the activation of specific developmental pathways. Importantly, this case emphasizes the critical need for thorough histopathological examination to detect microscopic non-seminomatous components in predominantly seminomatous tumors, as these can significantly alter prognosis and recurrence patterns.

Postpubertal-type teratoma is considered a non-SGCT derived from GCNIS. GCNIS-derived tumors are histologically and clinically divided into seminomas and non-seminomas, with the latter encompassing embryonal carcinoma, yolk sac tumor, trophoblastic tumor, and postpubertal-type teratoma [7,8]. Postpubertal-type teratoma is associated with abnormalities on the short arm of chromosome 12 (12p) [9]. Although postpubertal-type teratomas can be complicated by somatic malignancies, such as carcinoma and sarcoma, there were no morphologic features typical of teratoma with somatic malignancy in this particular case.

The CT and MRI results indicated a well-defined tumor near the left renal hilum, which suggested a possible late relapse of a testicular seminoma to the lymph nodes. Retroperitoneal teratomas are commonly found near the upper pole of the kidney, predominantly on the left side [10], and the localization of the mass in this case was consistent with this finding. Several differential diagnoses were also considered, including a neurogenic tumor, GCT (primary or secondary), and Castleman’s disease. Liposarcoma, which is considered to be a differential diagnosis, was excluded because there was no fat component in the mass observed by CT or MRI. The mass showed an increased FDG uptake on 18F-FDG PET/CT, which suggested the presence of malignant tissue components [11]; however, the radiological findings and the tumor marker levels did not narrow down the differential, and the diagnosis relied on the histological findings.

Teratomas are considered the most common type of GCT. Recurrence of teratomas preceded by seminoma is extremely rare; however, there are reports indicating a 35-40% chance of non-seminomas relapsing as postpubertal-type teratomas [12,13]. The resected tumor tissue, in this case, contained poorly differentiated neural tissue as well as cartilaginous and stratified ciliated columnar epithelium, which is characteristic of postpubertal-type teratoma. Because of the histological diagnosis of postpubertal-type teratoma of the resected retroperitoneal tumor in this case, we considered that the testicular tumor diagnosed as a seminoma six years earlier may have had a non-seminomatous component. Thus, it may have been a mixed GCT with both seminomatous and non-seminomatous components. After a histological analysis of the resected retroperitoneal tumor, the previously resected testicular tumor specimen was reexamined by a pathologist. As suspected, a small amount of embryonic carcinoma was found within the seminoma, suggesting that the postpubertal-type teratoma was a recurrence of a mixed GCT of GCNIS origin.

Microscopic detection of the non-seminomatous component can be challenging and may unintentionally remove the specimen from further evaluation. Therefore, when suspecting a recurrence of a GCNIS-derived GCT, it is important to consider the possibility of a background mixed GCT. Pathological diagnosis requires the preparation of numerous specimens to identify microscopic non-seminomatous components, which is often difficult during routine clinical practice. When a tumor of GCNIS origin is diagnosed, as in this case, efforts should be made to accurately ascertain its relationship to any past pathology.

This case highlights the critical importance of thorough pathological examination in testicular tumors. In light of our experience, it is worth emphasizing the key principles of effective sampling and diagnosis. Thorough sampling based on detailed observation of gross findings is crucial in pathological diagnosis. Specimens should be prepared from all tumor components, including areas with hemorrhage, cystic changes, and varying coloration. When elevated tumor markers are present, regardless of gross appearance, numerous specimens should be prepared to search for microscopic non-seminomatous components. Sharing accurate clinical information is essential to prevent sampling errors during sectioning. Additionally, the testicular parenchyma surrounding the tumor must always be sampled to assess for the presence of GCNIS [14].

The late recurrence, in this case, had a significant psychological impact on the patient. As is common in such cases, the patient was in the prime of his working life, and his quality of life was temporarily affected during treatment. This experience underscores the importance of clear communication with healthcare providers and the need for ongoing psychological support throughout the treatment process.

This case highlights the importance of extended follow-up, even for patients initially diagnosed with pure seminoma. We recommend imaging studies for at least 10 years post-treatment, with annual CT or MRI of the abdomen and pelvis, even in the absence of elevated tumor markers. During follow-up, clinicians should consider the possibility of recurrence if a new retroperitoneal mass appears, tumor markers rise, or unexplained abdominal or back pain persists. While surgery and chemotherapy remain the primary treatments, targeted therapies show promise for recurrent cases. One case report documented that KRAS mutations in metastatic tumors led to the use of anti-PD-1 therapy [15]. This approach may benefit patients with late recurrences or chemotherapy-resistant disease, highlighting the importance of molecular profiling. However, further research is needed to establish the efficacy of these emerging treatments.

Long-term vigilance is crucial in managing patients with testicular GCTs, even those initially diagnosed with stage I seminomas. There is a small possibility that such patients may actually have mixed GCTs, with non-seminomatous components potentially present despite meticulous histological examination. As demonstrated by our case, late recurrences can still occur despite careful monitoring. The insights gained from this experience have informed our approach to patient care, emphasizing the need for comprehensive management strategies. These should address not only the medical aspects but also the psychological and quality-of-life considerations associated with long-term surveillance and potential late recurrences. By maintaining a high index of suspicion and employing a holistic care approach, clinicians can better serve patients with a history of GCNIS-derived GCTs, ensuring optimal outcomes even years after initial treatment.

This case highlights several important clinical insights for managing testicular GCTs: (i) Microscopic detection of non-seminomatous components can be challenging, even with thorough pathological examination. When suspecting a recurrence of a GCNIS-derived GCT, it is crucial to consider the possibility of an initially undetected mixed GCT. (ii) Long-term follow-up beyond current guidelines may be necessary, as demonstrated by this late recurrence after six years. This emphasizes the need for individualized follow-up strategies in GCT management. (iii) Atypical recurrences may require reexamination of original pathology specimens, which can significantly impact diagnosis and treatment strategies. (iv) Advanced imaging techniques may not always provide definitive diagnoses in complex cases, necessitating more aggressive diagnostic approaches, including biopsy. (v) Multidisciplinary collaboration among urologists, radiologists, pathologists, and oncologists is vital in managing complex GCT recurrences.

## Conclusions

Reexamination of a six-year-old testicular tumor revealed a small amount of embryonal carcinoma within the seminoma, indicating that this postpubertal-type teratoma was a recurrence of a mixed GCT. These findings underscore the importance of sustained vigilance, comprehensive diagnostic approaches, and personalized long-term follow-up strategies in GCT management. By implementing these insights, clinicians may improve patient outcomes through more precise and individualized care.
